# Supplementary material for: Exciton polariton condensation from bound states in the continuum at room temperature
Source: Nat Commun. 2024 Apr 18;15:3345. doi: 10.1038/s41467-024-47669-8 (PMC11026397; doi:10.1038/s41467-024-47669-8)
Supplement: Supplementary file 1 — Supplementary information [file 41467_2024_47669_MOESM1_ESM.pdf]

Supplementary Information

Exciton Polariton Condensation from Bound States in the Continuum at Room Temperature

Xianxin Wu[1,2,#], Shuai Zhang[1,#], Jiepeng Song[3,#], Xinyi Deng[3], Wenna Du[1,2], Xin Zeng[1], Yuyang Zhang[1], Zhiyong Zhang[1,4], Yuzhong Chen[5], Yubin Wang[6], Chuanxiu Jiang[1,2], Yangguang Zhong[1], Bo Wu[7], Zhuoya Zhu[1,2], Yin Liang[3], Qing Zhang[3*], Qihua Xiong[5,6,8,9,*], and Xinfeng Liu[1,2,*]

[1]CAS Key Laboratory of Standardization and Measurement for Nanotechnology, National Center for Nanoscience and Technology, Beijing 100190, P. R. China

[2]University of Chinese Academy of Sciences, Beijing 100049, P. R. China

[3]School of Materials Science and Engineering, Peking University, Beijing 100871, P. R. China

[4]School of Physical Science and Technology, Inner Mongolia University, Hohhot 010021, P. R. China.

[5]Beijing Academy of Quantum Information Sciences, Beijing 100193, P. R. China

[6]State Key Laboratory of Low-Dimensional Quantum Physics and Department of Physics, Tsinghua University, Beijing 100084, P. R. China

[7]Guangdong Provincial Key Laboratory of Optical Information Materials and Technology, Institute of Electronic Paper Displays, South China Academy of Advanced Optoelectronics, South China Normal University, Guangzhou 510006, P. R. China.

[8]Beijing Innovation Center for Future Chips, Tsinghua University, Beijing 100084, P. R. China

[9]Frontier Science Center for Quantum Information, Beijing 100084, P. R. China

[*]E-mail: liuxf@nanoctr.cn; qihua_xiong@tsinghua.edu.cn; q_zhang@pku.edu.cn

[#]These authors contributed equally to this work.

Table of Content

Fig. S1. Morphologic characterizations of single-crystalline $CsPbBr_3$ microplatelets.
Fig. S2. Optical properties of $CsPbBr_3$ microplatelets and PhC lattices.
Fig. S3. Schematic of the fabrication and SEM images of the $CsPbBr_3$ PhC lattice.
Fig. S4. Schematic of the angle-resolved spectrum optical system.
Fig. S5. Simulated angle-resolved reflectance spectra of $CsPbBr_3$ PhC lattices with different number of periods (N).
Fig. S6. Extraction of polariton modes of $CsPbBr_3$ PhC lattice.
Fig. S7. Real-space images of the laser spot and the $CsPbBr_3$ PhC lattice emission.
Fig. S8. Evolution of emission spectra with different pump densities of $CsPbBr_3$ PhC lattices at different detunings in the vicinity of P_{th}.
Fig. S9. Pump density-dependent emission spectra of $CsPbBr_3$ PhC lattices at different detunings.
Fig. S10. Simulated excitonic weight of $CsPbBr_3$ PhC lattices with different detunings.
Fig. S11. Mode splitting resulting from the optical birefringence of the orthorhombic $CsPbBr_3$ single crystals.
Fig. S12. Quasi-continuous-wave optically pumped BIC polariton condensation at cryogenic temperatures.
Fig. S13. Spatial coherence of the $CsPbBr_3$ PhC lattice.
Fig. S14. Interference patterns of another sample in real space.
Fig. S15. Interference patterns of BIC polariton condensate emission in momentum space.
Fig. S16. Interference patterns in momentum space with the two arms misaligned.
Fig. S17. Real-space emission images of the miniaturized BIC polaritonic modes.
Fig. S18. Switching performance of different signal and gating conditions.
Note S1. Influence of the FIB etching process on the morphology and performance.
Fig. S19. Influence of thickness of $CsPbBr_3$ microplatelets.
Fig. S20. Simulation of angle-resolved reflectance spectra with different sample thicknesses.
Fig. S21. Influence of the depth of etching.
Fig. S22. Influence of the distortion during etching.
Fig. S23. Simulation of angle-resolved reflectance spectra of the PhC structure with partial etching and distortion.
Fig. S24. Simulation of angle-resolved reflectance spectra of the PhC structure with oblique sidewall.
Fig. S25. EDS elemental analysis spectrum.
Table S1. Elemental analysis of the etched region of $CsPbBr_3$ microplatelets.
Note S2. Numerical simulation of PhC lattice mode dispersion.
Fig. S26. Simulated mode dispersion of $CsPbBr_3$ PhC lattice.
Note S3. Polariton dispersion and BIC states at different detunings.
Fig. S27. Experimental and simulated angle-resolved reflectance spectra of $CsPbBr_3$ PhC lattices with different detunings.
Fig. S28. Angle-resolved PL spectra of $CsPbBr_3$ PhC lattices with different detunings.
Note S4. Time-resolved spectrum of BIC polariton condensation
Fig. S29. Time-resolved PL of the BIC polariton condensate.
Note S5. Numerical simulation of Fourier space distribution of PhC modes
Fig. S30. Simulated far-field distribution of electric field intensity at different wavelengths.

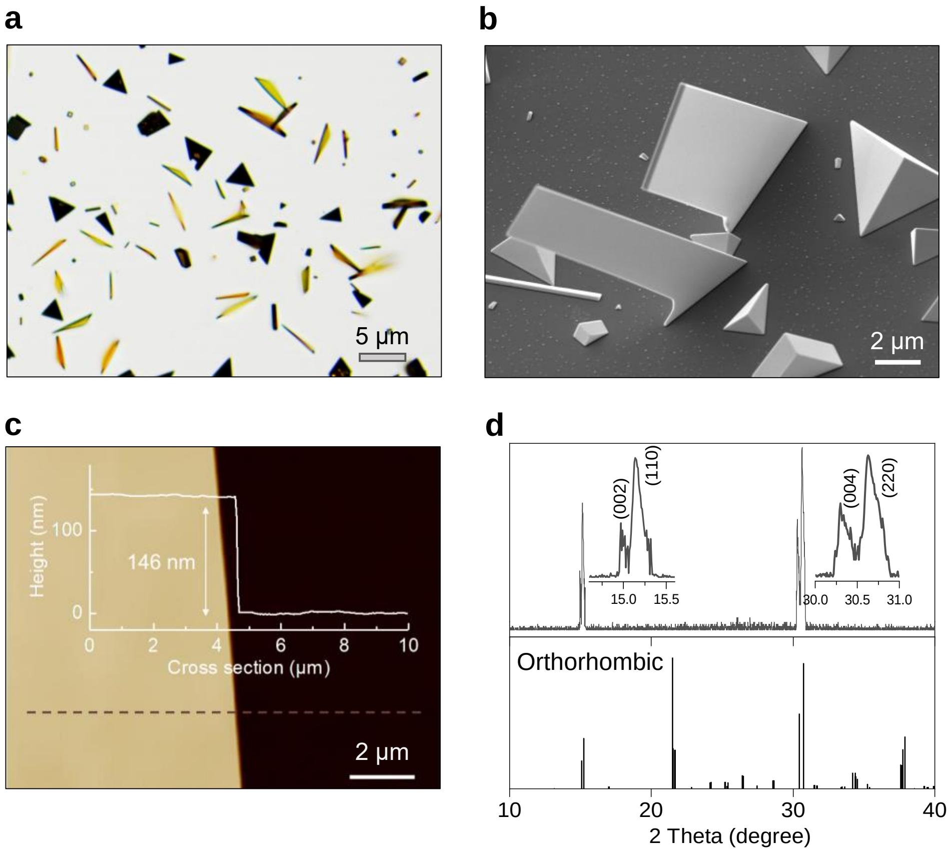

Fig. S1 | Morphologic characterizations of single-crystalline $CsPbBr_3$ microplatelets. a-b, Optical and scanning electron microscope (SEM) images of $CsPbBr_3$ microplatelets on Si/SiO_2 substrates, both the uniform color and smooth surface indicate a high crystal quality. **c,** The surface roughness and sample thickness were estimated to be around 1.9 nm and 146 nm in a scan area of 10 × 10 μm^2 by atomic force microscopy. **d,** X-ray diffraction patterns of the $CsPbBr_3$ microplatelets. The sharp peaks at 14.9°, 15.1°, 30.3°, and 30.6° correspond to (002), (110), (004), and (220) planes of orthorhombic phase, respectively, matching well with the standard structure reported in ref[1].

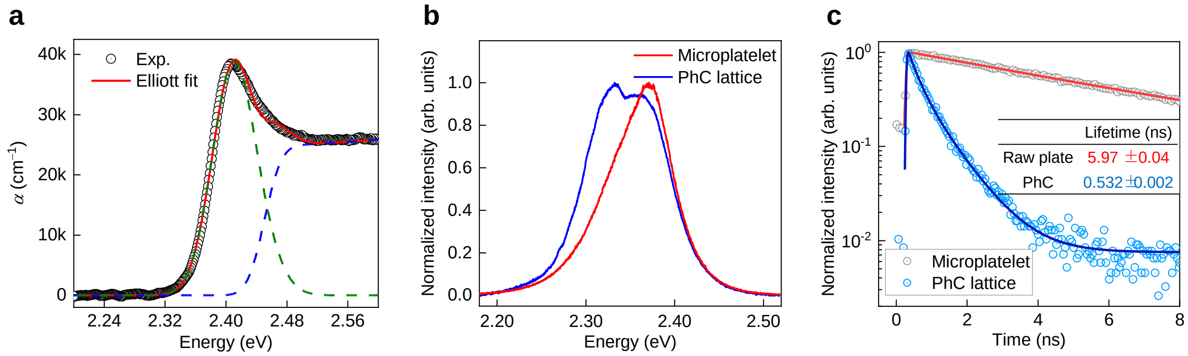

Fig. S2 | Optical properties of $CsPbBr_3$ microplatelets and PhC lattices. a, Measured reflection spectrum of $CsPbBr_3$ microplatelets. By fitting with Elliott theory (red solid line)[2, 3], the strong excitonic absorption peak suggests exciton energy be 2.41 eV and binding energy of ~40 meV, which is similar to the reported values on $CsPbBr_3$ microplatelets.[4-6] The fitted excitonic absorption (blue dotted line) suggests the narrow full width at half maximum (FWHM) of approximately 59.5 meV. **b,** Normalized photoluminescence (PL) spectra of the $CsPbBr_3$ microplate and photonic crystal (PhC) lattices obtained by pumping with a continuous-wave laser. PL emission from the $CsPbBr_3$ microplatelet exhibits an emission peak of 2.39 eV. The PL spectrum of the PhC lattice exhibits an energy redshift and a combination of multiple peaks, indicating the presence of coupling between the excitonic emission and PhC lattice cavity modes. **c,** Time-resolved PL spectra of $CsPbBr_3$ microplatelets and PhC lattices. The PL from $CsPbBr_3$ microplatelets shows a single-exponential decay with a lifetime of approximately 6.0 ns, which serves as evidence of its high quality and low trap density. In contrast, the PL from $CsPbBr_3$ PhC lattices displays a fast decay rate, which may be attributed to the modulation of the Purcell effect.

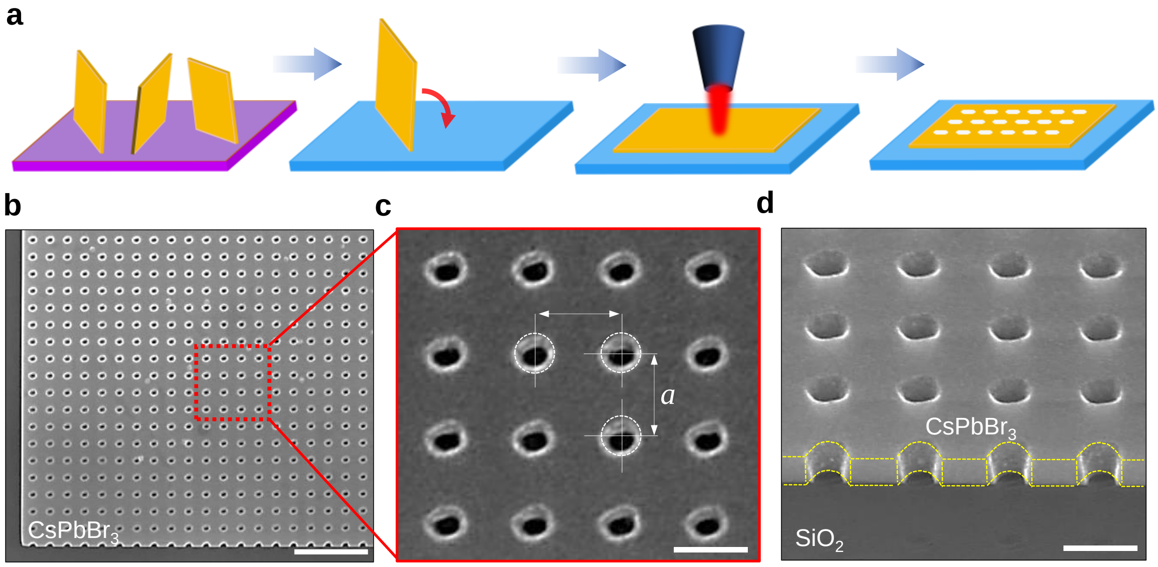

Fig. S3 | Schematic of the fabrication and SEM images of the $CsPbBr_3$ PhC lattice. a, Schematic diagram illustrating the growth, transfer, and focused ion beam (FIB) milling processes of single-crystalline $CsPbBr_3$ microplatelets. Out-of-plane $CsPbBr_3$ microplatelets were fabricated by the chemical vapor deposition method on silicon substrates. Subsequently, they were transferred onto the target substrate. FIB with optimized etching paths was applied to achieve precise carving of the air-hole array on the microplatelet. **b,** SEM image of the etched air holes on the $CsPbBr_3$ microplatelet, arranged in a square lattice. **c,** Zoomed-in image of (**b**). **d,** Tilt-view SEM image of the $CsPbBr_3$ PhC lattice The sharp sidewalls and smooth surface indicate negligible damage caused by FIB milling. Scale bars for (**b**)-(**d**) are 1.2 μm, 250 nm, and 250 nm, respectively.

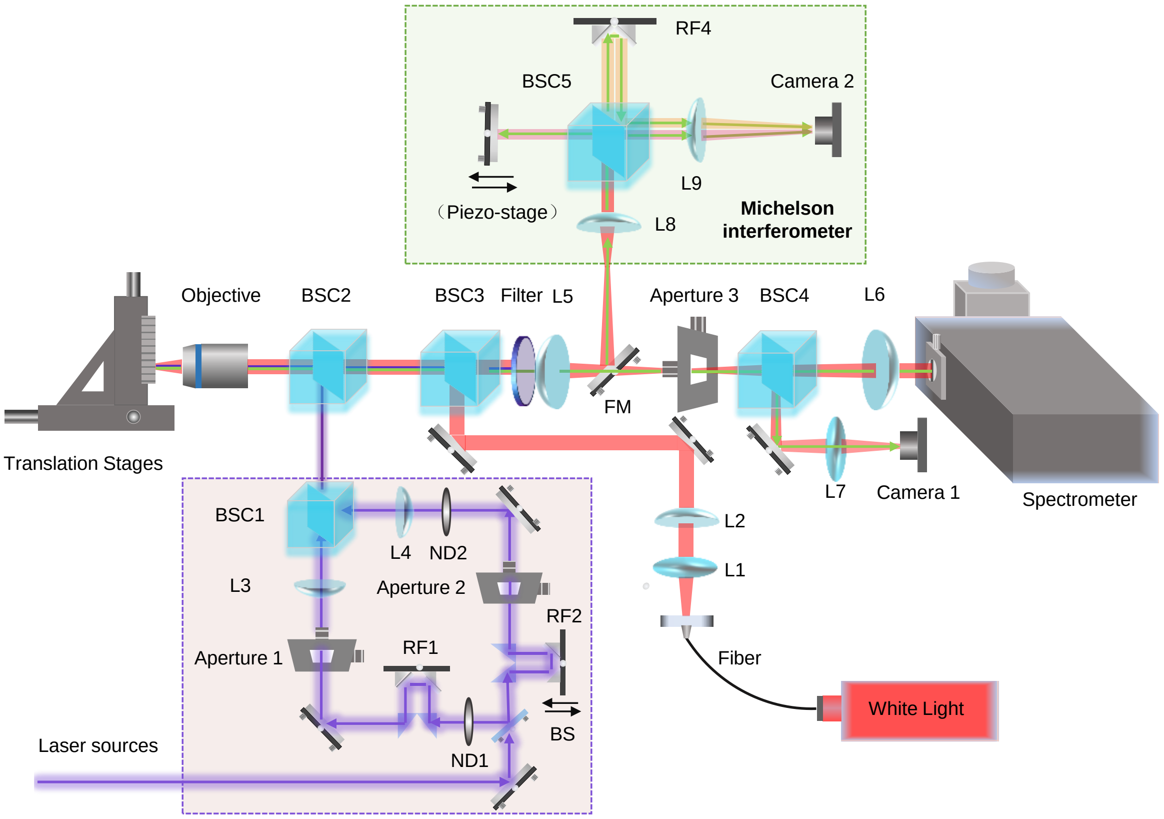

Fig. S4 | Schematic of the angle-resolved spectrum optical system. The laser sources are designed so that the sample can be excited by one or two laser spots with adjustable shape, density, and delay time. They are created by introducing the pump laser through a beam-splitter BS1 and retroreflectors RF1, 2 with a delay stage at RF2. The shape of two pump beams can be adjusted by a rectangular aperture (Aperture 1, 2) and lens (L3, 4). The pump density can be adjusted by neutral-density filters (ND1, 2). Laser sources and white light are introduced into the system by the beamsplitter cubes (BSC1, 2, 3). A 50× objective lens (Olympus MPLFLN, N.A. = 0.8) is used for focusing the light onto the sample. Plano-convex lenses (L5 and L6) are utilized for transforming between real-space and Fourier images. To simultaneously observe the Fourier image and its corresponding real-space image, a beamsplitter cube (BSC4) is incorporated into the light path. For the extraction of coherence profiles, the Michelson interferometer is integrated into the system, as labeled by the green dash lines based on the Fourier system. It consists of a retroreflector in the reference arm and a mirror mounted on a piezo stage in the delayed arm. The emission from the sample is directed onto the Michelson interferometer using a flip mirror FM. The lens of L8 can be assembled or removed to flexibly transfer between the real space or Fourier space coherence maps.

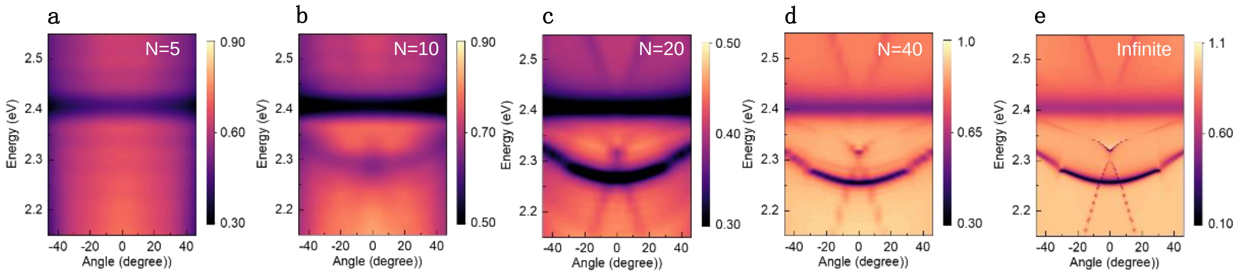

Fig. S5 | Simulated angle-resolved reflectance spectra of $CsPbBr_3$ PhC lattices with different number of periods (N). a-d, Simulated angle-resolved reflectance spectra of $CsPbBr_3$ PhC lattice with N values of 5, 10, 20, and 40, respectively. **e,** Simulated angle-resolved reflectance spectra of $CsPbBr_3$ PhC lattice with an infinite number of periods.

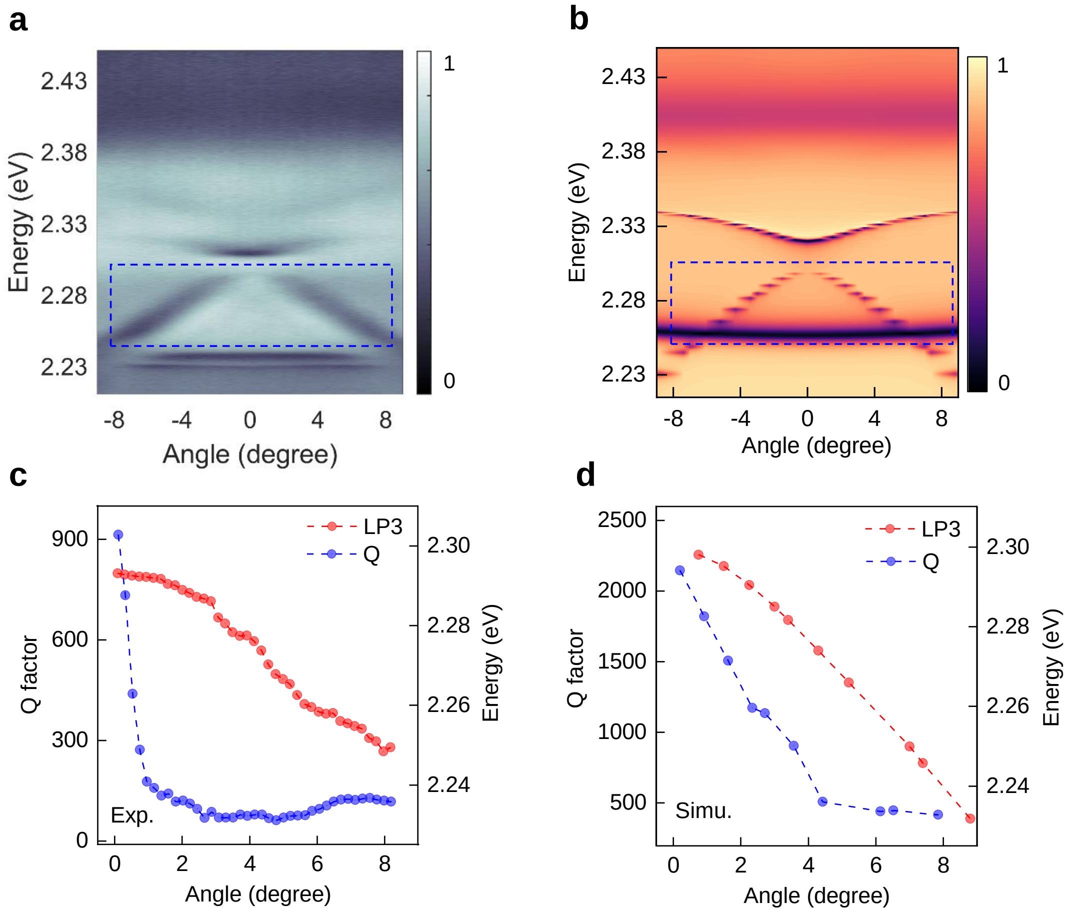

Fig. S6 | Extraction of polariton modes of $CsPbBr_3$ PhC lattice. **a,** Angle-resolved reflectance spectrum of the $CsPbBr_3$ PhC lattice taken from Fig. 1e of the main text. **b,** The corresponding numerical simulation of (**a**). The simulation data is reproduced from Fig. S24c in Note S2. The blue dotted regions are indicated as the LP3 polariton modes for extraction. **c,** The extracted energies and Q factors of the LP3 polariton mode near 0° in (**a**). **d,** The extracted energies and Q factors of the LP3 polariton mode near 0° in (**b**). The highest Q factor of 913 is observed in the vicinity of the dispersion maximum due to the presence of radiation-less BIC states established at the high-symmetry point.

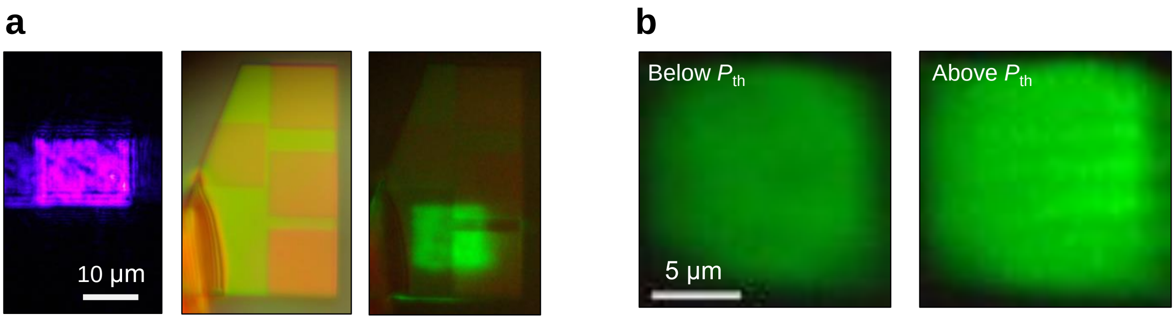

Fig. S7 | Real-space images of the laser spot and the $CsPbBr_3$ PhC lattice emission. a, From left to right: the optical images depict the laser spot, the $CsPbBr_3$ PhC lattices without laser pumping, and the $CsPbBr_3$ PhC lattices with partial laser pumping. The $CsPbBr_3$ PhC lattices exhibit higher emission intensity than the pristine microplatelet, indicating the enhancement effect achieved in the PhC lattice. **b,** The real-space PL images of the $CsPbBr_3$ PhC lattice under pulsed laser excitation with the pump density below and above P_{th}, respectively.

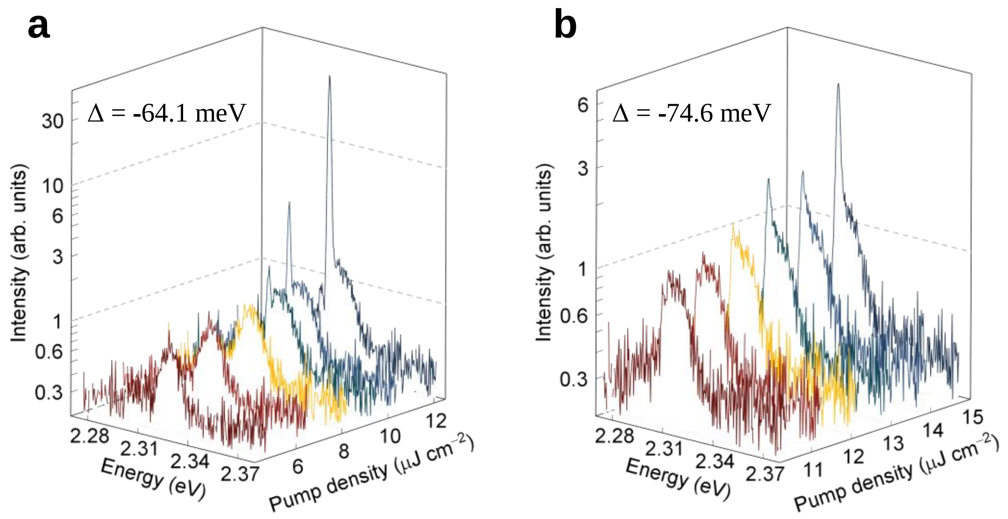

Fig. S8 | Evolution of emission spectra with different pump densities of $CsPbBr_3$ PhC lattices at different detunings in the vicinity of P_{th}. (**a**) Δ = −64.1 meV, (**b**) Δ = −74.6 meV.

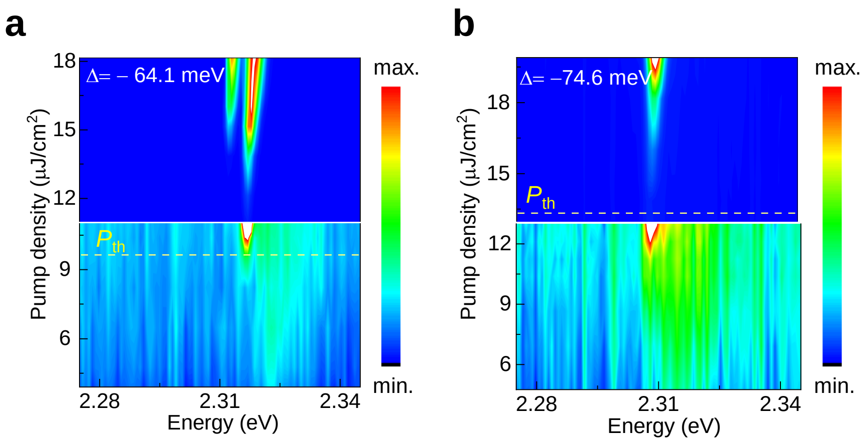

Fig. S9 | Pump density-dependent emission spectra of $CsPbBr_3$ PhC lattices at different detunings. a, Δ= −64.1 meV. **b,** Δ= −74.6 meV. To enhance visual clarity, the pseudocolor scale has been adjusted to accommodate two distinct ranges of pump density. The yellow dashed lines indicate the polariton condensation threshold.

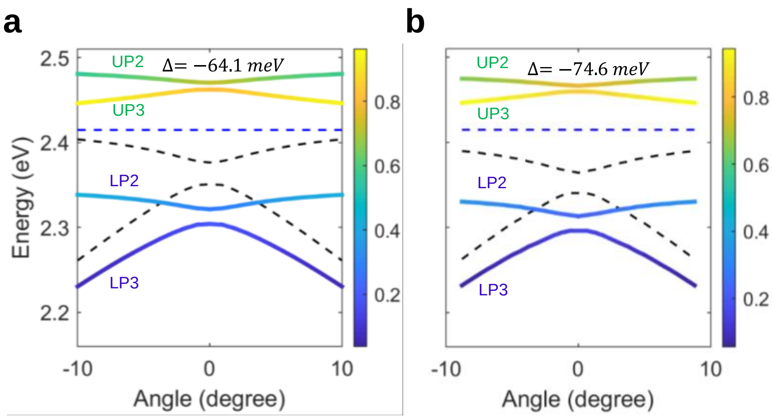

Fig. S10 | Simulated excitonic weight of $CsPbBr_3$ PhC lattices with different detunings. Polariton energy-angle dispersion calculated from the coupled harmonic oscillator model of $CsPbBr_3$ PhC lattices with $\Delta = -64.1$ meV (**a**) and -74.6 meV (**b**), respectively. The colors in the images represent a linear representation of the excitonic fraction for each mode, ranging from 0 (photon) to 1 (exciton).

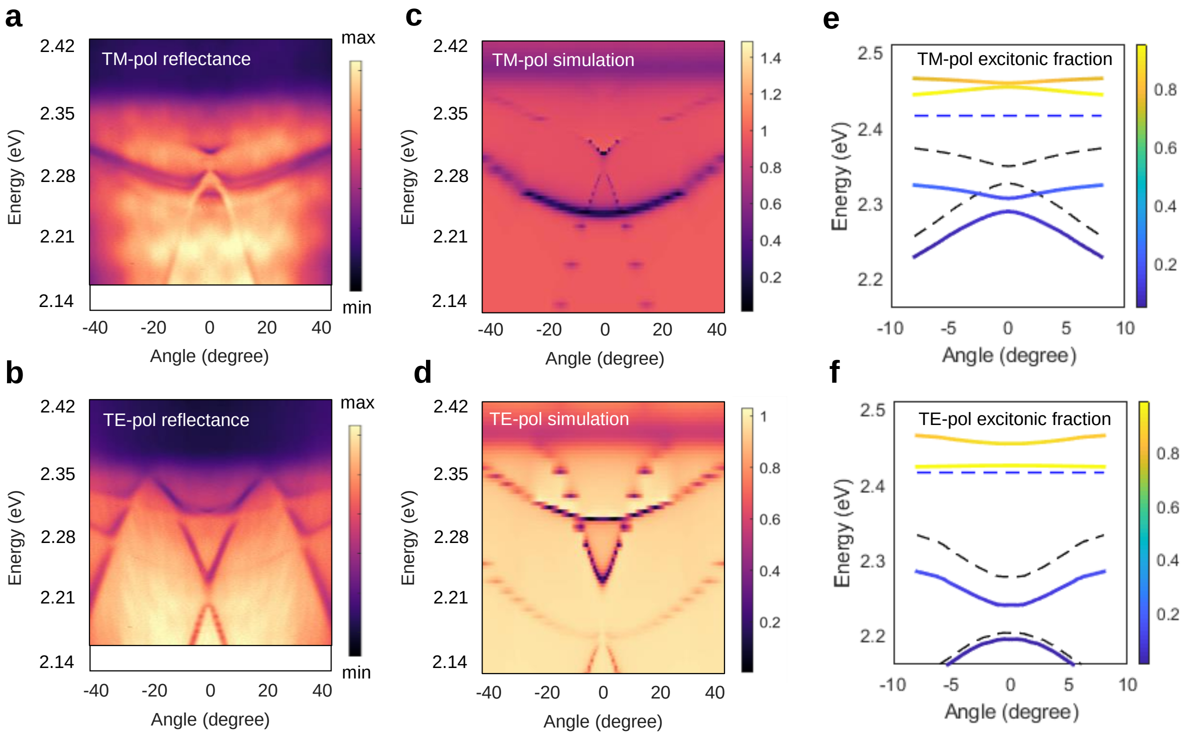

Fig. S11 | Mode splitting resulting from the optical birefringence of the orthorhombic $CsPbBr_3$ single crystals. a-b, Angle-resolved reflectance spectrum of the air-hole $CsPbBr_3$ PhC lattice. A linear polarizer was incorporated into the collection path to extract between emissions of TM-polarization (TM-pol, **a**) and TE-polarization (TE-pol, **b**), respectively. **c-d,** The corresponding simulations without considering the optical birefringence of $CsPbBr_3$. **e-f,** The corresponding simulated excitonic weights based on the coupled harmonic oscillator model. The colors in the images represent a linear representation of the excitonic fraction for each mode, ranging from 0 (photon) to 1 (exciton). The observation of BIC polariton condensation exclusively in the TM-polarization can be attributed to the longer lifetime of the TM-polarized state, stemming from a higher excitonic fraction (~16%), in contrast to the TE-polarized state (~1%).

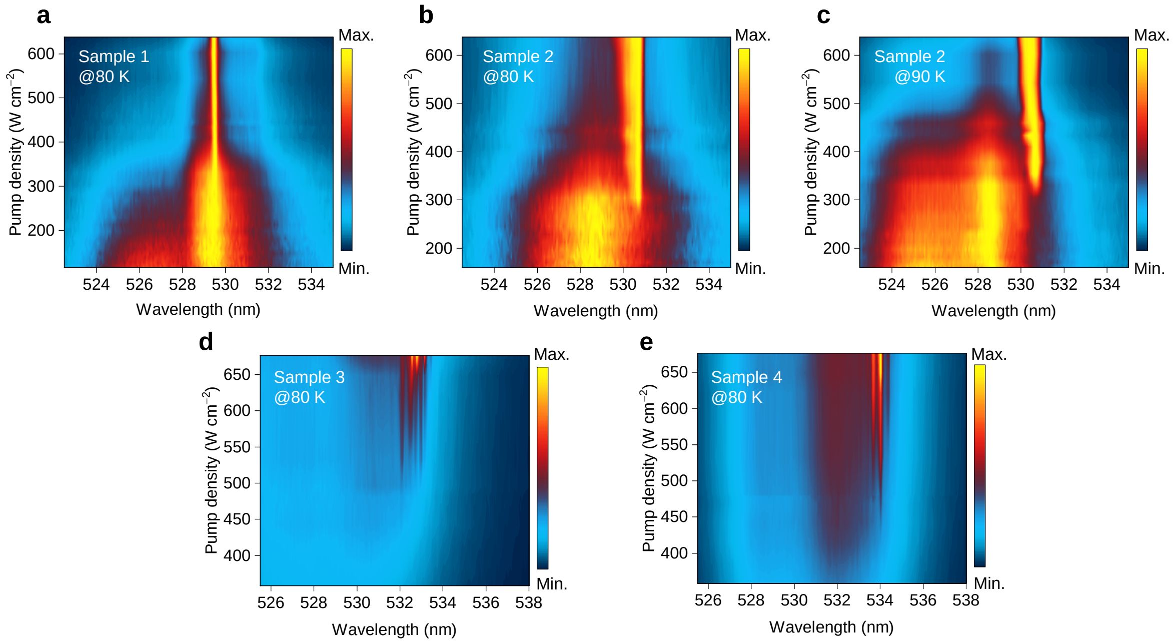

Fig. S12 | Quasi-continuous-wave optically pumped BIC polariton condensation at cryogenic temperatures. a-c, The 2D pseud-color mapping for PL spectra as the pump density increases for two $CsPbBr_3$ PhC lattices, sample 1 at 80 K (**a**), as well as sample 2 at 80 K (**b**) and 90 K (**c**). Notably, BIC polariton condensation could not be observed in sample 1 above 80 K and sample 2 above 90 K, indicating the potential for further improvement in sample quality. A clear transition from spontaneous emission to a condensate state was observed. The polariton condensation threshold of sample 2 at 90 K slightly increased compared to that at 80 K. **d-e,** The 2D pseud-color mapping for PL spectra as the pump density increases for two pristine $CsPbBr_3$ microplatelets.

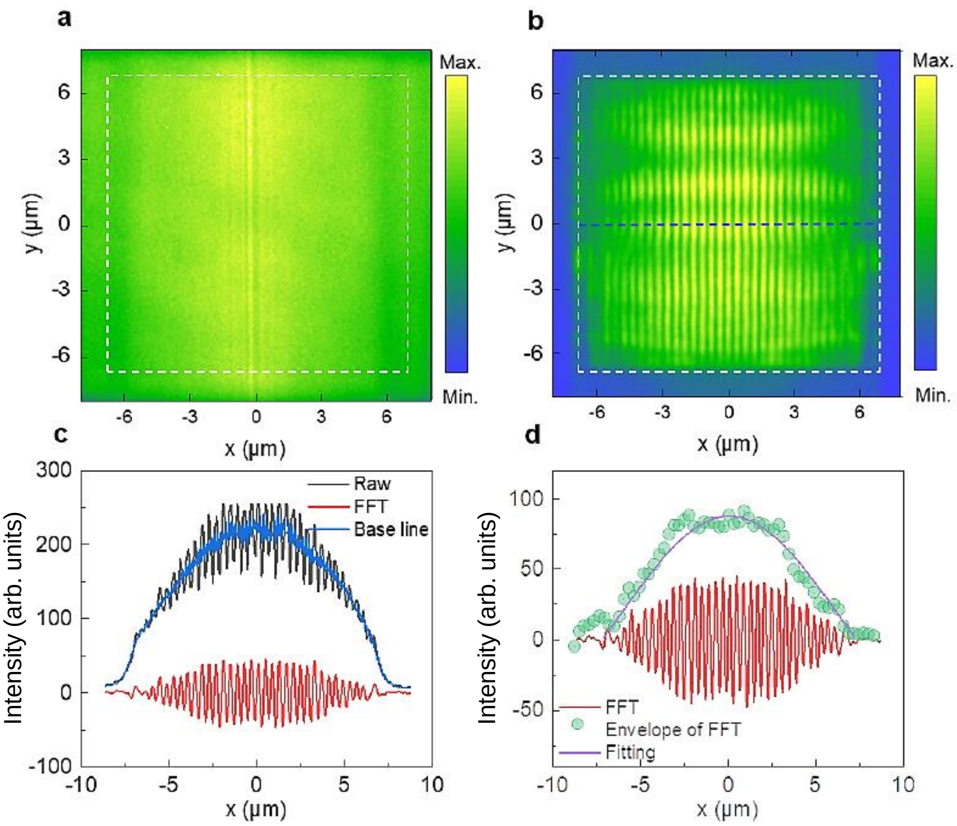

Fig. S13 | Spatial coherence of the $CsPbBr_3$ PhC lattice. a-b, Superposition of the real-space image and its inverted image for the $CsPbBr_3$ PhC lattice below and above P_{th}. In the absence of polariton condensation (**a**), the system exhibits a sharp coherence line with a correlation length below 1 μm, indicating that it remains in the thermal regime. When pumped above P_{th} (**b**), clear interference fringes emerge in the superposition region, suggesting a long-range spatial coherence. **c-d,** A spatial coherence distribution was derived from a cross-section of the interference pattern in (**b**). Through fast Fourier transform (FFT) and inverse fast Fourier transform (IFFT) processes, the pure interference signal (red line) was isolated from the baseline (blue line). The amplitude profile of the spatial coherence was then determined based on the envelopes of the interference signal. A spatial coherence length of 8.7 μm was obtained with a Gaussian function fitting.

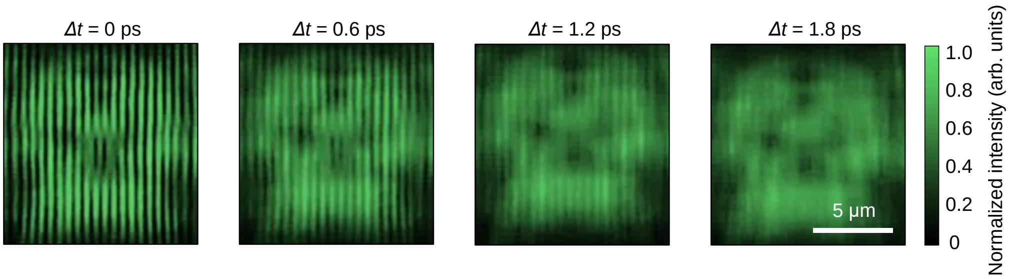

Fig. S14 | Interference patterns of another sample in real space. The patterns are acquired at Δt = 0, 0.6, 1.2, and 1.8 ps, respectively.

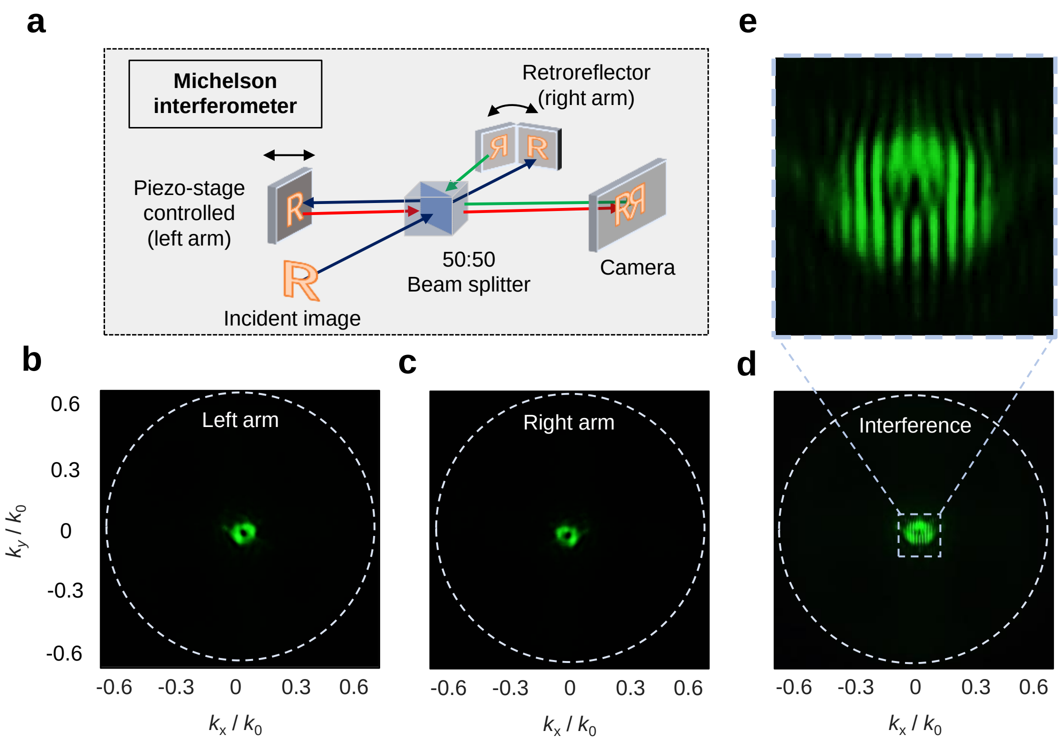

Fig. S15 | Interference patterns of BIC polariton condensate emission in momentum space. a, Schematic of the Michelson interferometer integrated into the Fourier system. The superposition of the original image and its inverted counterpart results in a mirror-symmetrical interference image. **b-c,** Back-focal plane (BFP) images of BIC polariton condensate emission collected from the left (**b**) and right arm (**c**) of the interferometer. **d,** Michelson interference pattern obtained by overlapping (**b**) and (**c**). **e,** Magnified interference pattern from (**d**), where two forks exhibiting a mirror-symmetric configuration are observed.

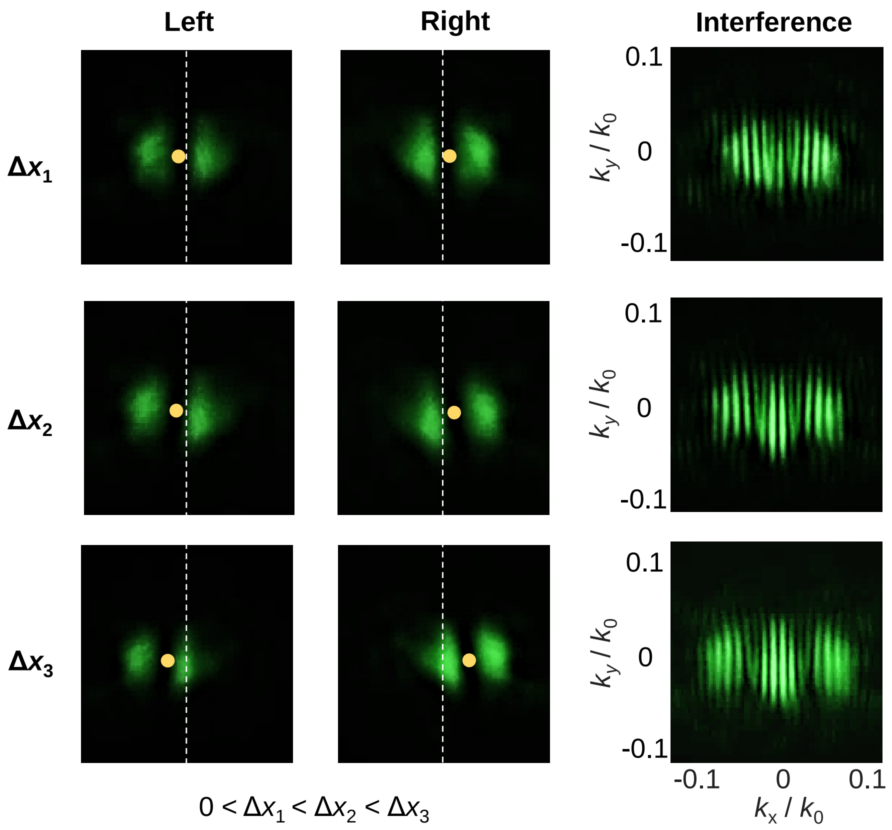

Fig. S16 | Interference patterns in momentum space with the two arms misaligned. BFP images of BIC polariton condensate emission collected from the left arm (first column), the right arm of the interferometer (second column), and their interference pattern (third column), with distinct misaligned distances ($0 < \Delta x_1 < \Delta x_2 < \Delta x_3$).

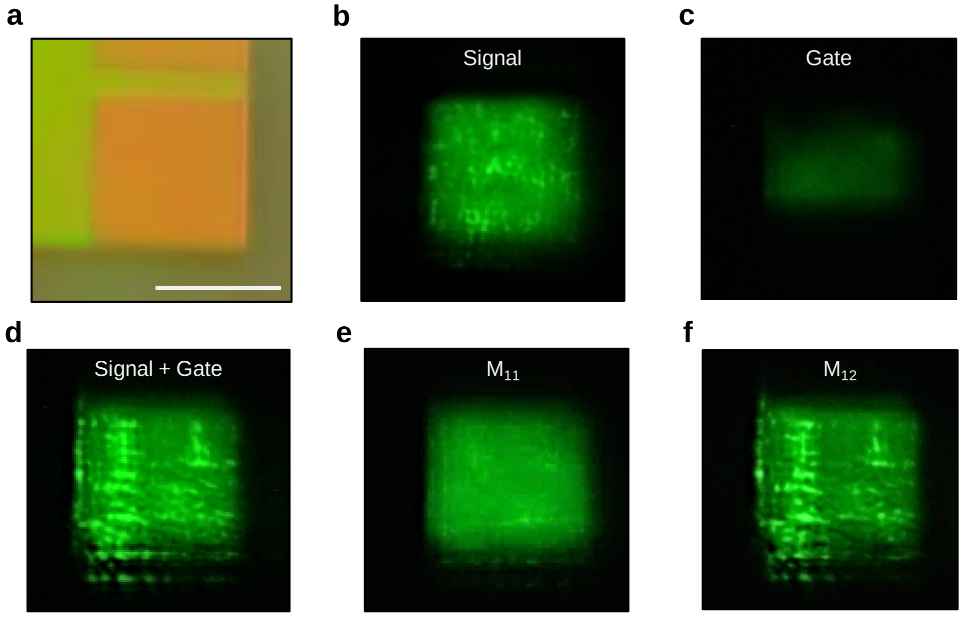

Fig. S17 | Real-space emission images of the miniaturized BIC polaritonic modes. a, Optical image of the measured $CsPbBr_3$ PhC lattice. Scale bar: 10 μm. **b-d,** Real-space PL image of the $CsPbBr_3$ PhC lattice under the excitation of the signal beam (~P_{th}, **b**), the gate beam (~0.2 P_{th}, **c**), and two beams together (**d**). **e-f,** Real-space PL image of the emission from the miniaturized BIC polaritonic modes M_{11} (**e**) and M_{12} (**f**).

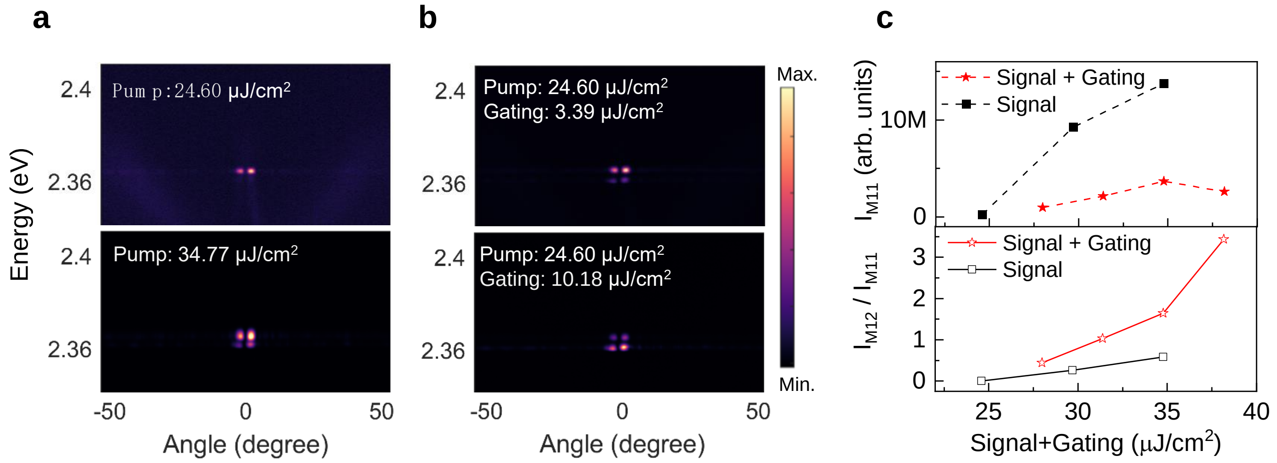

Fig. S18 | Switching performance of different signal and gating conditions. a, Angle-resolved emission of miniaturized BIC polaritonic modes under the signal beam of different pump densities. The emission is dominated at M_{11} mode. **b,** Under the pumping of the signal beam with fixed density and gating beam of varied density, the dominated emission is easily turned from M_{11} to M_{12} mode. **c**, Summary of the intensity of M_{11} (I_{M11}) and enhanced factor of M_{12} relative to M_{11} (I_{M12} / I_{M11}) at different pump densities of signal and gating beam.

Note S1. Influence of the FIB etching process on the morphology and performance

In our investigation of the influence of the FIB etching process on the morphology and performance of $CsPbBr_3$ PhC lattices, three aspects are concentrated here including the thickness of $CsPbBr_3$ microplatelets, etching depth, and the induced distortion. The influence of different lattice constants is used for precisely controlling the detuning and will be discussed in Note S2.

#1. Influence of thickness of $CsPbBr_3$ microplatelets.

$CsPbBr_3$ microplatelets of three different thicknesses were fully etched with the same lattice constants. The SEM images of etched PhC lattices are shown in Fig. S19a. Corresponding angle-resolved reflectance spectra in Fig. S19b indicate that the position of the BIC mode redshifts (more negative detuning) with the increase of thickness. Notably, as the thickness increases, there is a transition of BIC polariton condensation from LP3 to LP1 due to the much smaller detuning of LP3 (Fig. S19c).

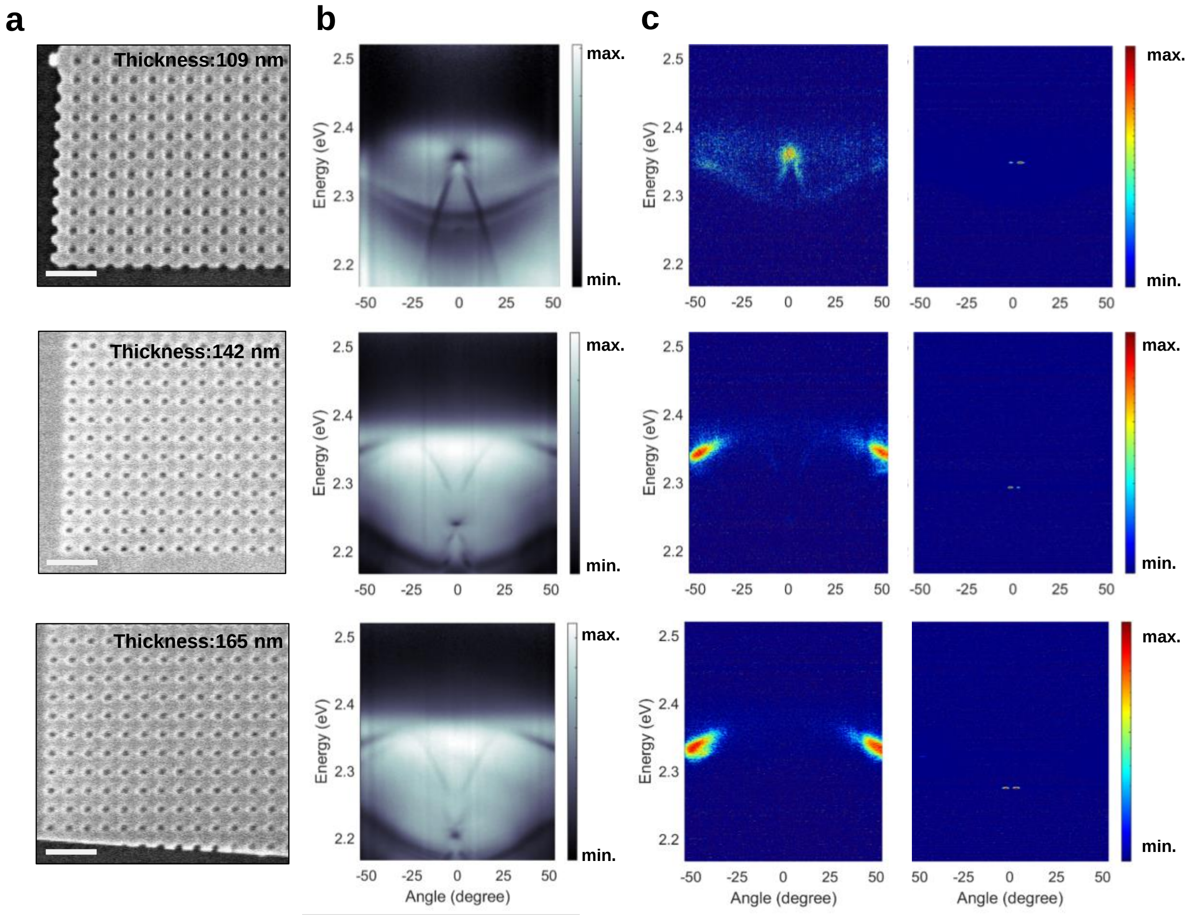

Fig. S19 | Influence of thickness of $CsPbBr_3$ microplatelets. a, SEM images of the $CsPbBr_3$ PhC lattice with different sample thicknesses for etching. Scale bar: 1 μm. **b,** Corresponding angle-resolved reflectance spectra respectively to (**a**). **c,** Angle-resolved PL spectra with the pump density below (left panel) and above P_{th} (right panel), respectively.

We made numerical simulations of PhC lattice mode dispersion (the simulation details are depicted in Note S2, similarly hereinafter) for samples with thickness l of 120 and 160 nm while keeping other parameters as constants (period a = 292 nm, and radius of etched hole r = 57 nm). The result of l = 140 nm is depicted as an example of simulation in Note S2 and hence is not shown here. The simulations in Figs. S20a-b imply that BIC polariton modes LP1, and LP3 redshift as the sample thickness increases, which is consistent with experimental results in Fig. S19. We note that the Q factor of LP3 increases greatly at a small angle when l = 140 nm, while this trend is much weakened when l = 160 nm and not applicable when l = 120 nm. Considering the actual range of thickness of $CsPbBr_3$ microplatelets, the samples with thickness l ~145 ± 5 nm are ideal for FIB etching.

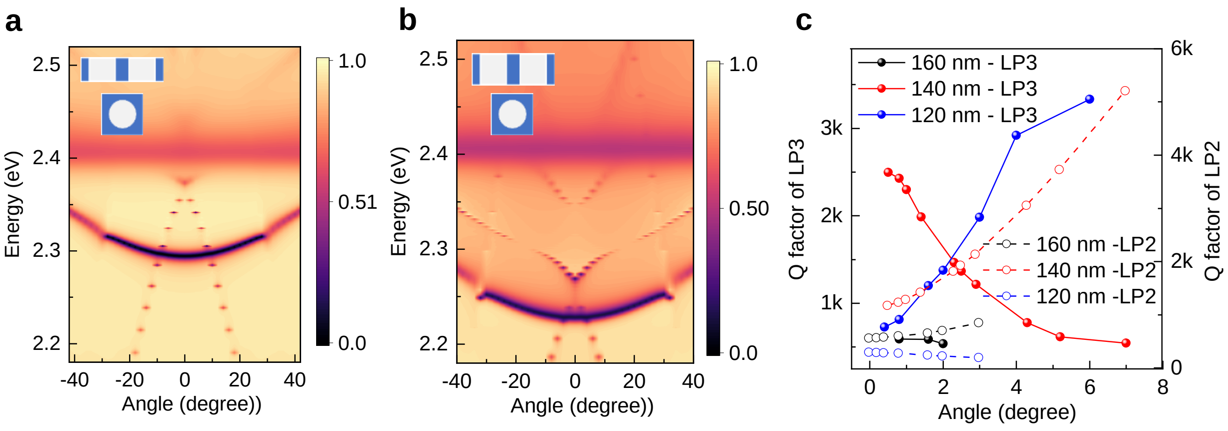

Fig. S20 | Simulation of angle-resolved reflectance spectra with different sample thicknesses. a, l = 120 nm. **b,** l = 160 nm. Inserts are corresponding sketch maps of the side view (upper) and top view (lower) of $CsPbBr_3$ PhC lattices. **c,** The extracted Q factors of bright polariton modes LP2 and corresponding dark modes LP3 for samples with different l.

#2. Influence of etching depth and distortion during the etching process.

Regarding the etching depth, we observed that it has a notable impact on the mode dispersion of the BIC polaritons (Fig. S21). Compared with the fully etched sample with similar parameters, the partially etched sample exhibits single parabolic dispersion and is blurred at large angles. We considered that the partial etching would influence its mirror image in the Z-axis direction. The inversion symmetry and mirror-flip symmetry are all broken causing the degradation of the theoretical infinite-lifetime BIC state and is replaced by a leaky resonance[7]. Although under high-power excitation, we could still observe the lasing character at zero momentum, the emission cannot be ascribed to the condensation of the BIC state now, but related to the leakage mode at TE polarization.

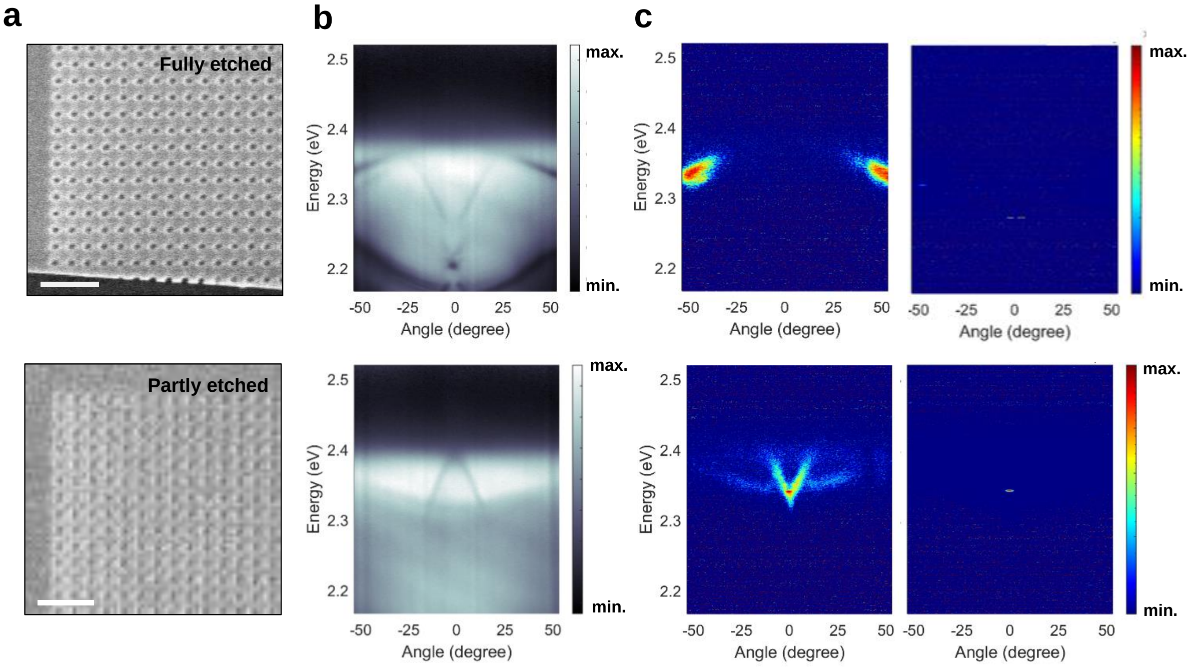

Fig. S21 | Influence of the depth of etching. a, SEM images of the $CsPbBr_3$ PhC lattice with excessive (upper) and insufficient (lower) etching depths. Scale bar: 1 μm. **b,** The corresponding angle-resolved reflectance spectra. **c,** Corresponding angle-resolved PL spectra with the pump density below (left panel) and above P_{th} (right panel).

If the ion beam during the etching process is oblique to the sample, distortion can be generated as an elliptical-shaped hole, as shown in Fig. S22a. Compared with the round hole, there is a symmetry reduction at Γ point of the first Brillouin zone, i.e. C_4^z symmetry is broken while retaining C_2^z symmetry. Hence, the symmetry-protected BICs are no longer allowed. Due to the topological charge conservation, BIC before symmetry reduction cannot suddenly disappear and should split into separated topological charges at off Γ points. Since the structure still preserves mirror symmetry, the split BICs are constrained in the TM direction[8]. In the angle-resolved PL spectra, two laser-like emissions appear at symmetric angles around the zero momentum, which coincide with the two symmetric subbranches in the angle-resolved reflectance spectra (Figs. S22b-c). The BIC characteristics at off Γ points need further investigation and will not be discussed here.

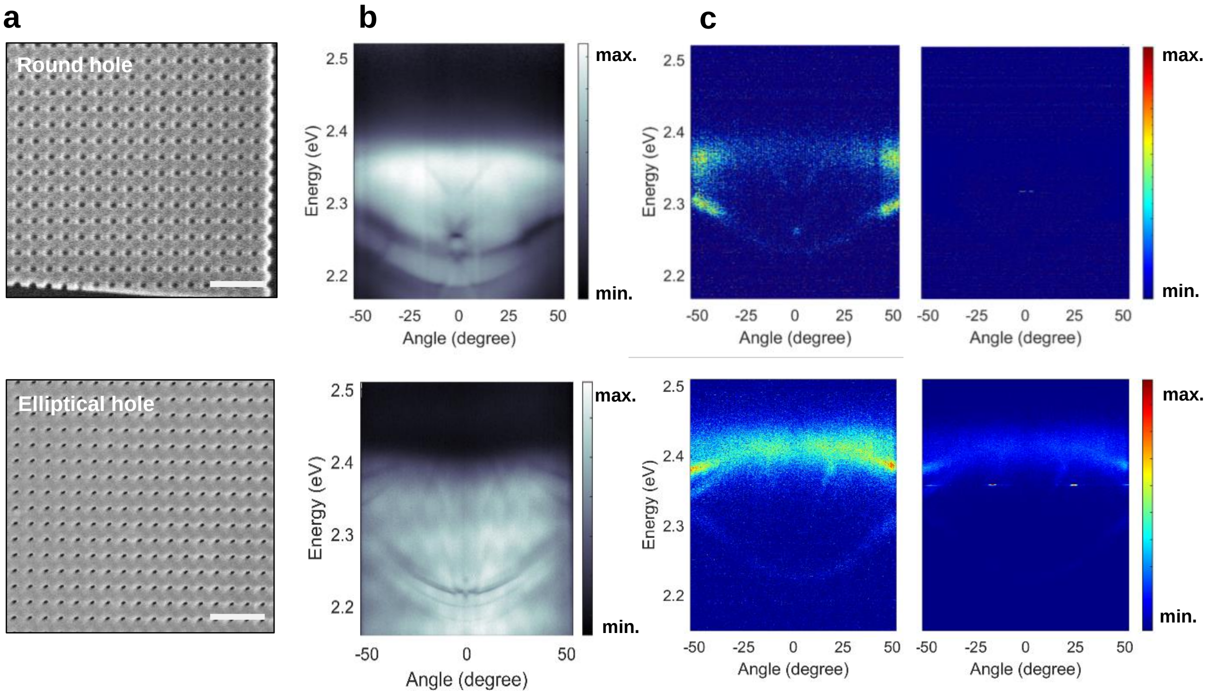

Fig. S22 | Influence of the distortion during etching. a-b, SEM images of the $CsPbBr_3$ PhC lattice with different distortion statuses during etching. Scale bar: 1 µm. **c-d,** Angle-resolved reflectance spectra corresponding to (**a**) and (**b**). **e-f,** Angle-resolved PL spectra with the pump density below (left panel) and above P_{th} (right panel) corresponding to (**a**) and (**b**).

The corresponding numerical simulations of etching depth and distortion are provided in Fig. S23. In Fig. S23a, the structure comprises cylindrical holes that are incompletely etched through the microplatelet. resulting in a limited hole depth of 70 nm. Conversely, Fig. S23b showcases elliptical holes with a semi-minor axis of 28.5 nm and a semi-major axis of 57 nm. All other parameters remain consistent with the simulation example described in Note S2. Notably, neither of these structures exhibits a BIC state at zero momentum, despite the dominance of parabolic modes, which aligns with the experimental results.

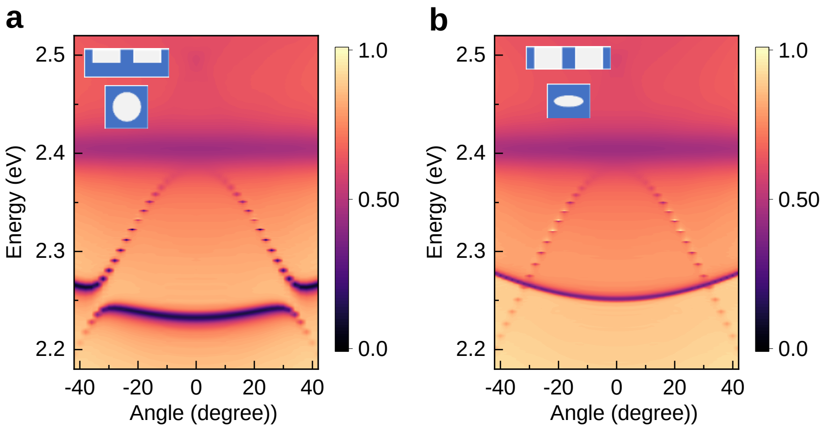

Fig. S23 | Simulation of angle-resolved reflectance spectra of the PhC structure with partial etching and distortion. a, Etched holes with a depth of 70 nm. **b,** Elliptical holes with their semi-minor axis of 28.5 nm and semi-major axis of 57 nm. Inserts are corresponding sketch maps of the side view (upper) and top view (lower) of $CsPbBr_3$ PhC lattices.

#3. Influence of oblique sidewall during the etching process.

Due to the challenges associated with altering the sidewalls of the etched holes while keeping other parameters unchanged, our analysis primarily focuses on investigating the influence of oblique sidewalls using numerical simulations. Specifically, we employ a bowl-shaped configuration with etched holes characterized by an upper radius of 57 nm and a lower radius of 30 nm. The simulation results presented in Fig. S24a suggest that the incline of the sidewall does not affect the location of the BIC state at Γ point of the first Brillouin zone. Despite the absence of mirror-flip symmetry in this structure, the inversion symmetry remains intact.

We compare the *Q* factors of oblique sidewalls with those of vertical sidewalls (as exemplified in the simulation example described in Note S2), as shown in Fig. S24b. The results reveal a significant increase in the *Q* factor of LP3 when structures are formed at a small angle in both cases. Nevertheless, this increase is somewhat attenuated in the structure featuring oblique sidewalls, indicating a potential decrease in the *Q* factor.

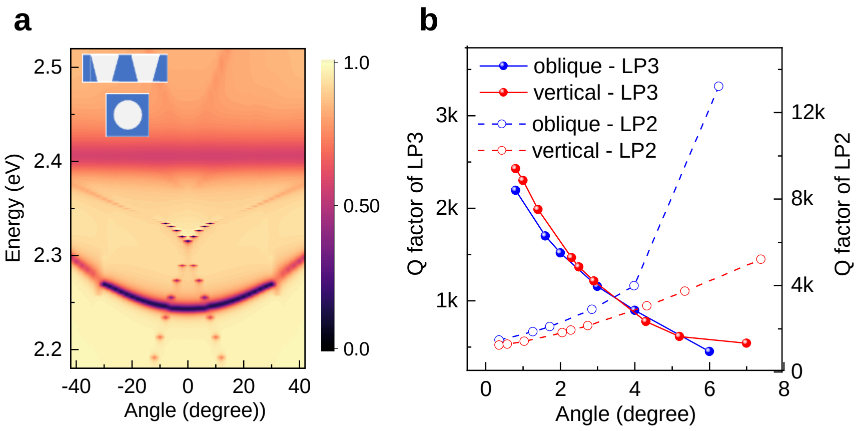

Fig. S24 | Simulation of angle-resolved reflectance spectra of the PhC structure with oblique sidewall. a, Bowl-shaped etched holes with an upper radius of 57 nm and a lower radius of 30 nm. Inserts are corresponding sketch maps of the side view (upper) and top view (lower) of $CsPbBr_3$ PhC lattices. **b,** The extracted *Q* factors of bright polariton modes LP2 and corresponding dark modes LP3 at different morphologies of the sidewall.

#4. Other possible factors during the etching process.

We observed rough surface features near the etched holes in the SEM images depicted in Fig. S3, ascribing to ion beam etching. These residues introduced by the etching process can increase coupling with nearby radiative states by scattering, posing a drawback to the *Q* factors of BICs. To mitigate such disadvantages during the etching process, it is crucial to confine the residues exclusively to the boundary of the etched hole, while ensuring they do not persist on the unetched surface or obstruct the hole. To address this concern, we employed the FEI Nova 200 NanoLab FIB system and positioned the sample stage at an inclination angle of 52°. This arrangement ensured that the ion beam

source was incident vertically on the sample, resulting in sharper sidewalls and fewer residues. Concurrently, the electron beam source was obliquely incident on the sample, allowing continuous observation of the etching state throughout the process. By adopting this approach, we minimized the influence of residues as much as possible.

To address any potential concerns regarding Galium ion contamination resulting from the etching process, we performed energy dispersive spectroscopy (EDS) elemental analysis of the etched sample, as shown in Fig. S25 and Table S1. The obtained results reveal a minimal presence of gallium ions. Notably, in the etching process, we deliberately utilized a very low ion beam current (<10 pA), and ensured a short residence time (maximum of 2 ms), effectively mitigating any substantial gallium ion contamination in the sample.

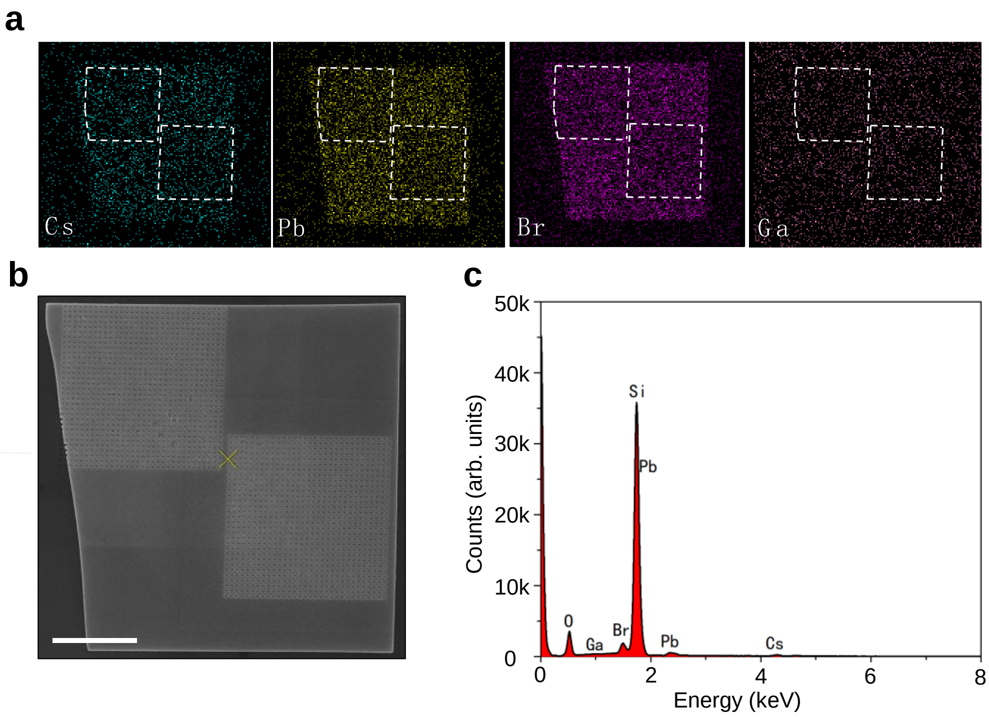

Fig. S25 | EDS elemental analysis spectrum. a, Psedu-color EDS images of Cs, Pb, Br, and Ga elements in uniformly distributed $CsPbBr_3$ microplatelet, respectively. The regions of white dotted lines represent etched areas. **b,** The corresponding SEM image. Scale bar: 5 μm. **c,** The content of each element in the entire scanning area.

Table S1 | Elemental analysis of the etched region of $CsPbBr_3$ microplatelets.

Element	Weight (%)	Atoms (%)
O	19.12	34.23
Si	59.24	60.42
Cs	5.28	1.14
Pb	7.47	1.03
Br	9.13	3.28
Ga	-0.24	-0.10

Note S2. Numerical simulation of PhC lattice mode dispersion

We investigated the mode dispersions of perovskite PhC lattices using numerical simulations based on the full-wave electromagnetic finite-difference time-domain (FDTD) method. Our simulation model comprised a square $CsPbBr_3$ microplatelet placed on a SiO_2/Si substrate with air holes drilled on it, covered by a polymethyl methacrylate (PMMA) layer. To construct the simulation model, we derived the geometric parameters for the unit cell from SEM images. The wavelength-dependent complex permittivity of $CsPbBr_3$ was imported from ref[9]. For a trade-off between computational efficiency and accuracy, we set the mesh size of the simulation model to 2 nm. Two field monitors were strategically placed above and below the air-hole structure to record the reflection and transmission signals. A plane wave source was utilized to inject light into the simulation region, and Bloch boundary conditions were applied in the x and y directions to calculate the response of the entire periodic system by simulating just a single one-unit cell.

For the simulation of angle-resolved spectra, we tuned the plane wave sweeping along the tilt angles. Switching between transverse-electric (TE) and transverse-magnetic (TM) detection is realized by changing the polarization angle of the plane wave. As described in ref[9-11], the TM modes exhibit a confined electric field primarily within the high-index regime, specifically within the perovskite air-hole PhC system. Conversely, the TE modes predominantly reside within the low-index regime. To realize a stronger interaction between PhC photons and perovskite excitons, a significant spatial overlap between the electrical field and perovskite is necessary. Therefore, we focus on the TM-polarized mode dispersion within our system.

To accurately model the behavior of the perovskite PhC lattice, we separated the complex permittivity into its constituent parts: the continuum absorption band and the excitonic component. By focusing on the pure absorption band component, we conducted simulations of the PhC modes without excitonic contributions, represented by the red line in Fig. S26a. The calculated parameters are set as thickness l = 140 nm, period a = 292 nm, and radius of etched hole r = 57 nm. The angle-resolved reflectance spectra of the perovskite PhC lattice, both with and without excitons, are depicted in Figs. S26b-c. Notably, the angle-resolved reflectance spectrum in Fig. S26c displays intriguing mode dispersion with an anti-crossing behavior around the exciton energy, suggesting a strong coupling between the PhC photons and excitons.

Meanwhile, the simulated angle-resolved reflectance spectrum presented in Fig. S26c closely aligns with the experimental results shown in Fig. 1e, confirming the reliability and accuracy of our simulation approach. To further quantify the strength of the interaction, we extracted the mode dispersion from the angle-resolved reflectance spectrum of the complex permittivity without excitons

(Fig. S26b), which enables us to perform subsequent calculations based on the coupled harmonic oscillator model.

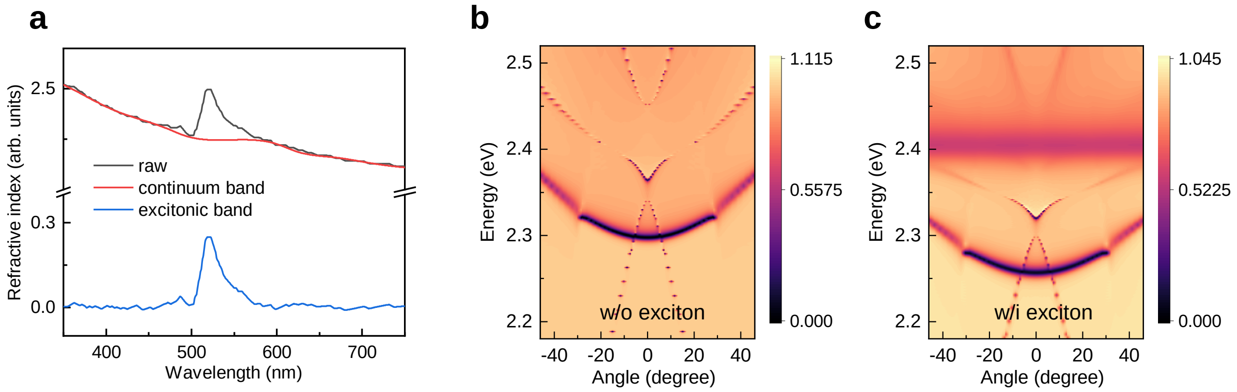

Fig. S26 | Simulated mode dispersion of $CsPbBr_3$ PhC lattice. a, Refractive index of $CsPbBr_3$ fitted with continuum and excitonic bands. The raw index data was extracted from ellipsometer results[9]. To obtain the intrinsic cavity mode dispersions and prevent calculation results from the influence of excitons part in the medium, the refractive index of $CsPbBr_3$ was separated into two components above. **b,** Mode dispersions of perovskite PhC lattice without excitons involved were acquired by collecting the signals of transmission and reflection signals. **c,** Mode dispersions of perovskite PhC lattice with excitons involved.

The coupled harmonic oscillator model was introduced to explain the formation of exciton polariton in this perovskite PhC lattice. The system can be described by a four-coupled-oscillator Hamiltonian:[12, 13]

$$H_p(k)=\begin{bmatrix} E_X-i\Gamma_X & V_b & 0 & 0 \\ V_b & M_b(k)-i\Gamma_b(k) & 0 & 0 \\ 0 & 0 & E_X-i\Gamma_X & V_d \\ 0 & 0 & V_d & M_d(k)-i\Gamma_d(k) \end{bmatrix} \tag{1}$$

Here, $M_b(k)-i\Gamma_b(k)$ and $M_b(k)-i\Gamma_b(k)$ are the energy-momentum dispersion of bright and dark cavity modes, $E_X-i\Gamma_X$ is the excitonic part, V_b and V_d are the coupling strength for bright/dark modes. Considering that the anti-node of the bright/dark mode corresponds to the node of the other one, a given exciton cannot be coupled simultaneously to both photonic modes. Approximation was made that half of the exciton only coupled to the dark mode and the rest part coupled to the bright mode. Therefore V_b □ V_b □ $\Omega/2$, where Ω is the Rabi splitting energy[12]. Calculation of the eigenvalues of each 2 × 2 region of H_p corresponds to UPs/LPs with a dark (upper left) or bright (lower right) nature:

$$E_{b,d,L}=\frac{M_{b,d}(k)+E_X-i(\Gamma_X+\Gamma_{b,d}(k))}{2}-\sqrt{\frac{(\Delta_{b,d}+i(\Gamma_X-\Gamma_{b,d}(k)))^2}{4}+V^2} \tag{2}$$

$$E_{b,d,U}=\frac{M_{b,d}(k)+E_X-i(\Gamma_X+\Gamma_{b,d}(k))}{2}+\sqrt{\frac{(\Delta_{b,d}+i(\Gamma_X-\Gamma_{b,d}(k)))^2}{4}+V^2} \tag{3}$$

Here $\Delta_{b,d} = M_{b,d}(k) - E_X$ is the detuning energy. The dispersion and linewidth of M_b and M_d are imported from simulated PhC mode dispersion as discussed in Fig. S25. Exciton energy and linewidth are set as 2.14 eV and 60 meV based on fitting of excitonic absorption in Fig. S2. In this way, the anti-crossing of both bright and dark modes can be interpreted. The eigenvectors of UPs/LPs are expressed as:

$$H_p(k) \cdot \begin{pmatrix} X_{L,U} \\ C_{L,U} \end{pmatrix} = E_{L,U}(k) \cdot \begin{pmatrix} X_{L,U} \\ C_{L,U} \end{pmatrix} \tag{4}$$

Hopfield coefficients $C_{L,U}(k)$ and $X_{L,U}(k)$ satisfy $\left|C_{L,U}(k)\right|^2 + \left|X_{L,U}(k)\right|^2 = 1$ and indicates the photonic or excitonic weight. For the LPB, they are given by:

$$C_L(k) = -\frac{V}{\sqrt{V^2 + \left(E_L(k) - M_{b,d}(k)\right)^2}}, \quad X_L(k) = \frac{M_{b,d}(k) - E_L(k)}{\sqrt{V^2 + \left(E_L(k) - M_{b,d}(k)\right)^2}} \tag{5}$$

Note S3. Polariton dispersion and BIC states at different detuning

The detuning of cavity mode relative to the excitonic resonance can be precisely controlled by the lattice period *a*. We define the detuning as the offset of dark Mode 3 relatives to excitonic resonance (E_x = 2.41 eV). Fig. S27a shows the angle-resolved reflectance spectra of $CsPbBr_3$ PhCs with different lattice periods. From left to right, *a* = 290, 295, 300, and 300 nm, respectively. The thickness of the left three samples is *l* = 140 nm while the sample on the right side is *l* = 150 nm. The yellow and red solid lines are polariton branches fitted by the coupled harmonic oscillator model. The corresponding cavity Mode 2 and Mode 3 can thus be deduced. The detuning becomes more negative as the period increases. Although the thickness of the sample can also result in different detuning, it is difficult to arbitrarily adjust due to technical limitations. Fig. S27b is the corresponding numerical simulation results of polariton dispersion by FDTD methods.

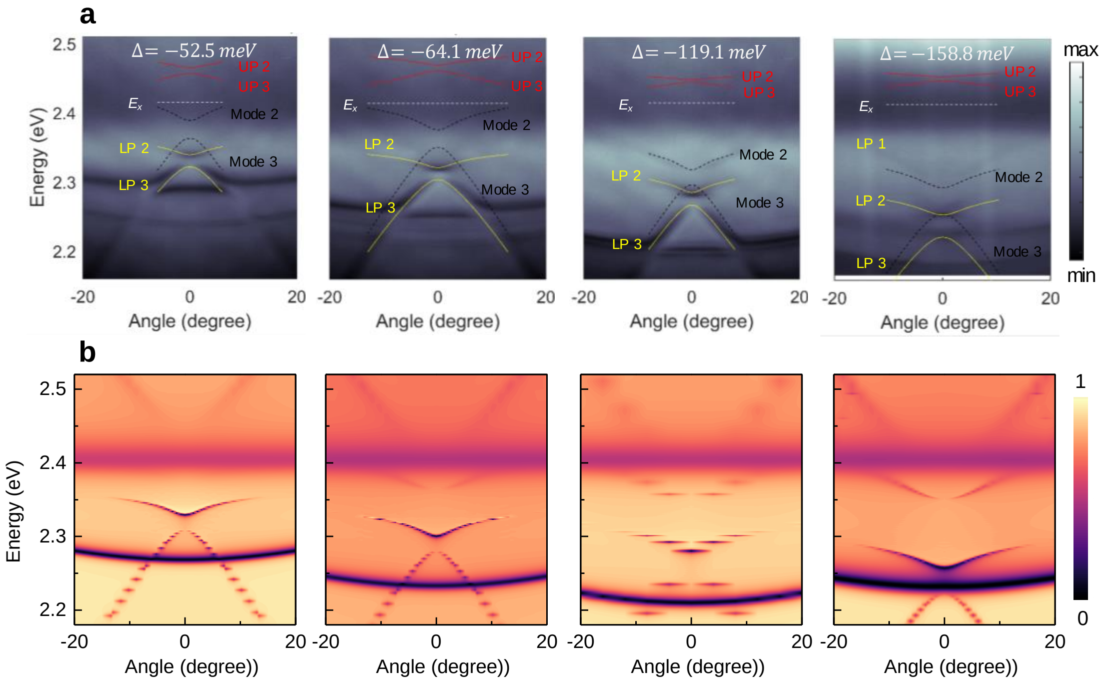

Fig. S27 | Experimental and simulated angle-resolved reflectance spectra of $CsPbBr_3$ PhC lattices with different detunings. a, Experimental angle-resolved reflectance spectra of $CsPbBr_3$ PhC lattice with Δ of −52.5, −64.1, −119.1, and −158.8 meV, respectively. The solid lines are fitted polariton branches and black dashed lines are the corresponding photonic modes. **b,** The corresponding simulated results.

Fig. S28 presents the corresponding angle-resolved PL spectra below and above P_{th}. From left to right, Mode 3 exhibits an increasingly negative detuning, leading to a redshift of the LP3. However, when the detuning is too negative, polariton condensates at this BIC energy become unfavorable due to the amplified loss before reaching the final condensation. Consequently, lasing actions may occur at alternative polariton branches or even other BIC modes with higher energy, such as LP1 at Δ = −158.8 meV.

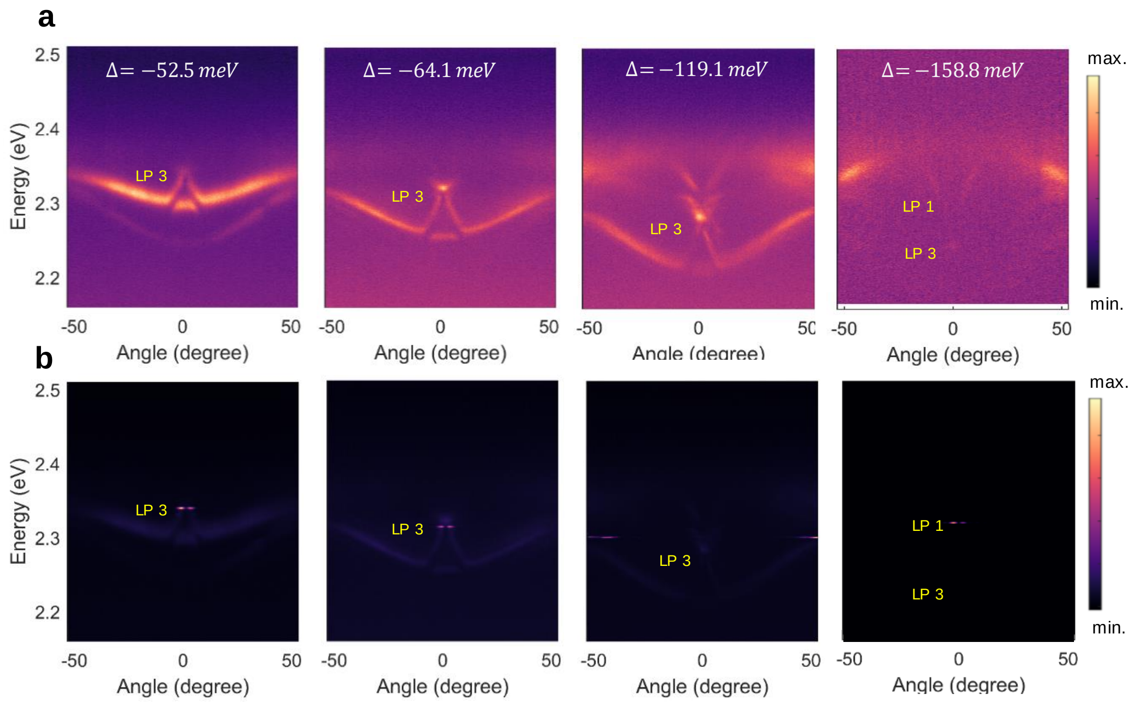

Fig. S28 | Angle-resolved PL spectra of $CsPbBr_3$ PhC lattices with different detunings. a, Under pulsed laser excitation with a pump density below P_{th}. **b,** Under pulsed laser excitation with a pump density above P_{th}. The marked LP1 and LP3 indicate the position of the BIC state at these modes.

Note S4. Time-resolved spectra of BIC polariton condensates

The transient PL spectra of BIC polariton condensates were conducted utilizing a homebuilt ultrafast optical Kerr gating system. Three samples with different detunings, corresponding to different BIC polaritonic mode energies, were measured. The steady-state angle-resolved PL spectra of these samples below and just above P_{th} are shown in Fig. S29a and Fig. S29b, respectively. As we progress from Sample No. 1 to No. 3, the BIC mode energy increases from 2.318 to 2.340 eV. To examine the time evolution of PL intensity and energy, the signals from different angles are combined and presented in Fig. S29c. At 1.1 P_{th}, we observed a continuous redshift of BIC polariton condensate energy and a gradual decay of PL intensity after reaching its maximum. However, for Sample No. 1, an unusual plateau around 1.6 ps is observed. Given its significant negative detuning, further investigation is required to elucidate this behavior, which could be related to an inefficient relaxation pathway. Moreover, we extracted the time-resolved intensity curves, as depicted in Fig. S29d. The curves for Sample No. 2 and No. 3 can be fitted with a mono-exponential decay function, yielding time constants of 2.57 and 3.07 ps, respectively. These obtained lifetimes are slightly larger but comparable to the coherence time measured by the Michelson interferometer (2.14 ± 0.16 ps). Note that the coherence time and lifetime of BIC polariton condensation can be influenced by various factors, including pump density and detuning, among others, which may vary depending on different parameters

For example, at 1.6 P_{th}, the relaxation of Sample No. 3 is accelerated, followed by a rapid decay before 2.4 ps and a slower decay thereafter (Fig. S29c). Fig. S29e compared the time-resolved intensity curves of the same energy at these two pump densities. At 1.1 P_{th}, fitting the mono-exponential decay function yields a time constant of 2.45 ps. However, when the pump density increased to 1.6 P_{th}, the fast component exhibited a time constant of only 1.27 ps, while the slower component showed a larger time constant of 2.87 ps. The saddle point at 2.48 ps suggests the decay of the fast mode and delayed generation of the slower mode. The decrease in the lifetime of the same BIC mode (2.45 ps to 1.27 ps) density is consistent with the decrease of coherence time at higher pump density. Additionally, considering the observed miniaturized BIC polaritonic modes at high pump density (Fig. S18), the dynamics of these two components can be explained by the increased polariton−polariton interaction at 1.6 P_{th}, which promotes the relaxation between different miniaturized BIC polaritonic modes.

Furthermore, the BIC polariton lifetime can be expressed as the inverse to the population decay rate, that is[14], $\gamma_{LP} = |X|^2 \cdot \gamma_{nr} + (1 - |X|^2) \cdot \gamma_C$. Here, γ_C is the out-coupling rate of a cavity photon, γ_{nr} is the nonradiative decay rate of an exciton, and $|X|^2$ is the excitonic fraction. A practical quality factor $<10^4$ is reasonable near BIC state energy, resulting in a cavity photon lifetime of approximately 2.5

ps. It should be noted that the quality factor obtained from the angle-resolved reflectance spectra may underestimate the exact cavity quality factor. On the other hand, for $CsPbBr_3$ perovskites excited by femtosecond pulsed laser at room temperature, the excitonic non-radiative recombination processes may involve polaron-assisted energy transfer, Auger recombination, and exciton-exciton annihilation, *etc*. Hence, in our case, we should not ignore the cavity photon out-coupling part considering the limited cavity quality factor in practice. Then, considering a $|X|^2$ of ~0.20 and an excitonic non-radiative lifetime of ~60 ps, a polariton lifetime of ~3.1 ps could be estimated, which agrees with our experimental results.

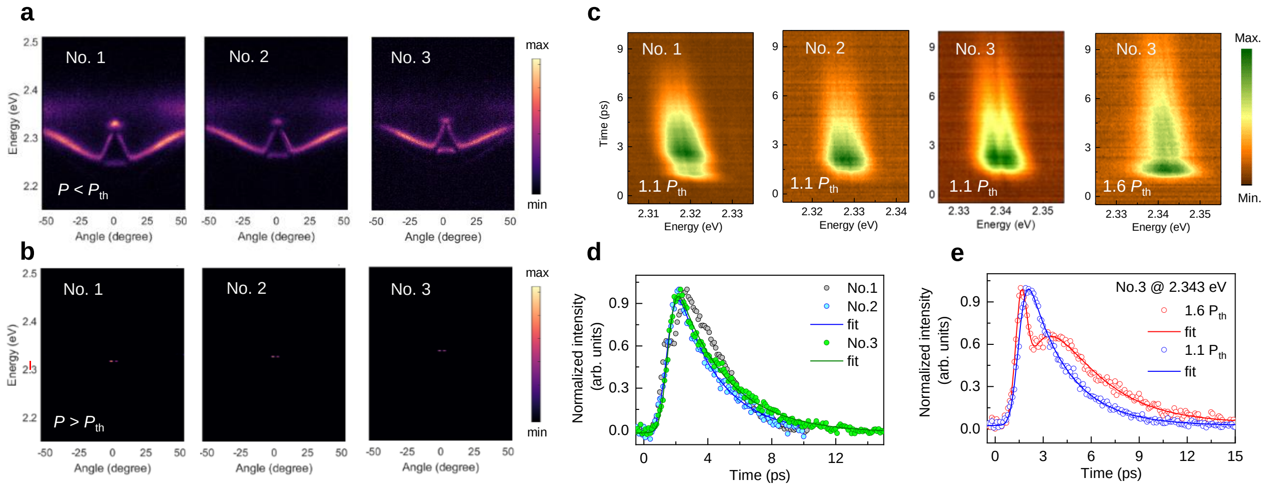

Fig. S29 | Time-resolved PL of BIC polariton condensates. a, Angle-resolved PL spectra of the measured $CsPbBr_3$ PhC lattice with different detunings. From left (No. 1) to right (No. 3), the energy of BIC polariton mode increases due to weaker negative detuning. **b,** The corresponding polariton condensation of these BIC polariton modes, which is ready for time-resolved PL measurements. **c,** Time-resolved PL emission of BIC polariton condensates at pump densities of 1.1 P_{th} (No. 1-3) and 1.6 P_{th} (No. 3), respectively. Continuous red-shift can be observed in these measurements. **d,** The normalized time-resolved intensity profiles at 1.1 P_{th} extracted from (**c**). The decay region can be fitted by the mono-exponential decay function. **e,** The normalized time-resolved intensity profiles of No. 3 at 1.6 P_{th}. As the pump density increases from 1.1 P_{th} to 1.6 P_{th}, The intensity profiles can be fitted by a mono-exponential or biexponential decay function.

Note S5. Numerical simulation of Fourier space distribution of PhC modes

The far-field emission of the $CsPbBr_3$ PhC lattice was simulated by the 3D FDTD method. The geometrical parameter was set as periodicity a = 292 nm, radius of etched hole r = 57 nm, thickness h = 140 nm. Due to the limitation of computing performance, the simulated region was limited as an area consisting of 40 × 40 unit cells, and the mesh grid was set as 5 nm × 5 nm × 10 nm. Two incoherent electrical dipoles with *s* and *p* polarization were arranged at the center of the simulating object[15]. The near-field monitor was set slightly above the PhC surface to record the electromagnetic field. Far-field distribution was obtained by the built-in far-filed projector. Since the PL emission of $CsPbBr_3$ PhC covers the wavelength mainly from 515 to 540 nm, we take the far-field projection containing multiple wavelengths into the final simulation. Each of the far-field projections can be viewed as a slice of the electromagnetic field at a constant wavelength[16]. Some of these far-field projections of electric field $|E|^2$ are shown in Fig. S30. Then we sum all the slices together, as is shown in Fig. 4c of the main text. The simulated result is mainly satisfied with the experiment in Fig. 4a of the main text, including the elliptical-shaped photonic bands along k_x and k_y directions, and the dark region around these bands. The BIC polariton condensate emission pattern in Fig. 4b of the main text exhibits the donut shape with a much smaller size than the overlapped region of elliptical-shaped photonic bands along k_x and k_y directions. This can be partly explained by the simulated far-field projection at 526 nm in Fig. S30. At this wavelength, a circular electromagnetic field pattern is observed at the vicinity of the Γ-point, which is qualitatively smaller than the overlapped region of elliptical-shaped photonic with high energy and large momentum (e.g., far-field projection at 522 nm and 524 nm). However, the BIC polariton condensates around the Γ-point dominate the emission pattern, *i.e.*, emissions of the same wavelength at other momentum coordinates are suppressed, which is not reproduced in this simulation.

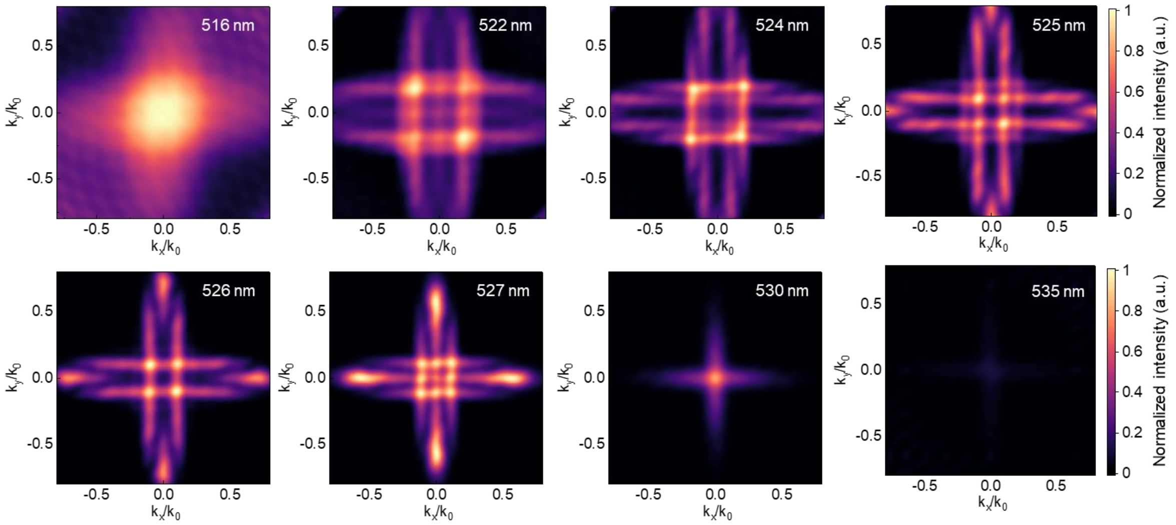

Fig. S30 | Simulated far-field distribution of electric field intensity at different wavelengths.

References

1. Eaton, S.W. et al. Lasing in robust cesium lead halide perovskite nanowires. *Proc. Natl. Acad. Sci. U.S.A.* **113**, 1993-1998 (2016).
2. Elliott, R.J. Intensity of optical absorption by excitons. *Phys. Rev.* **108**, 1384-1389 (1957).
3. Righetto, M. et al. Hot carriers perspective on the nature of traps in perovskites. *Nat. Commun.* **11**, 2712 (2020).
4. Zhang, Q. et al. High-quality whispering-gallery-mode lasing from cesium lead halide perovskite nanoplatelets. *Adv. Funct. Mater.* **26**, 6238-6245 (2016).
5. Zhang, S. et al. Trapped exciton–polariton condensate by spatial confinement in a perovskite microcavity. *ACS Photonics* **7**, 327-337 (2020).
6. Su, R. et al. Observation of exciton polariton condensation in a perovskite lattice at room temperature. *Nat. Phys.* **16**, 301-306 (2020).
7. Hsu, C.W. et al. Observation of trapped light within the radiation continuum. *Nature* **499**, 188-191 (2013).
8. Kang, M. et al. Merging bound states in the continuum by harnessing higher-order topological charges. *Light-Sci. Appl.* **11**, 228 (2022).
9. Huang, C. et al. Ultrafast control of vortex microlasers. *Science* **367**, 1018-1021 (2020).
10. Wu, M. et al. Bound state in the continuum in nanoantenna-coupled slab waveguide enables low-threshold quantum-dot lasing. *Nano Lett.* **21**, 9754-9760 (2021).
11. Heilmann, R., Salerno, G., Cuerda, J., Hakala, T.K. & Torma, P. Quasi-BIC mode lasing in a quadrumer plasmonic lattice. *ACS Photonics* **9**, 224-232 (2022).
12. Lu, L. et al. Engineering a light–matter strong coupling regime in perovskite-based plasmonic metasurface: quasi-bound state in the continuum and exceptional points. *Photonics Res.* **8**, A91-A100.
13. Ardizzone, V. et al. Polariton Bose–Einstein condensate from a bound state in the continuum. *Nature* **605**, 447-452 (2022).
14. Deng, H., Haug, H. & Yamamoto, Y. Exciton-polariton Bose-Einstein condensation. *Rev. Mod. Phys.* **82**, 1489-1537 (2010).
15. Chen, Y. et al. Metasurface integrated monolayer exciton polariton. *Nano Lett.* **20**, 5292-5300 (2020).
16. Regan, E.C. et al. Direct imaging of isofrequency contours in photonic structures. *Sci. Adv.* **2**, e1601591 (2016).
